# Assessing health impacts of the December 2013 Ice storm in Ontario, Canada

**DOI:** 10.1186/s12889-016-3214-7

**Published:** 2016-07-11

**Authors:** Nikhil Rajaram, Karin Hohenadel, Laera Gattoni, Yasmin Khan, Elizabeth Birk-Urovitz, Lennon Li, Brian Schwartz

**Affiliations:** Occupational Medicine Residency Program, 4th Fl. Shuter, St. Michael’s Hospital, 30 Bond Street, Toronto, ON M5B 1X1 Canada; Ontario Ministry of Labour, 505 University Avenue, Toronto, ON M7A 1 T7 Canada; Public Health Ontario, 480 University Avenue, Toronto, ON M5G 1 V2 Canada; Dalla Lana School of Public Health, University of Toronto, 155 College Street, Toronto, ON M5T 3 M7 Canada; Division of Emergency Medicine, Department of Medicine, University of Toronto, Toronto, ON Canada; Public Health and Preventive Medicine Residency Program, University of Toronto, 155 College Street, Toronto, ON M5T 3 M7 Canada

**Keywords:** Ice storm, Freezing rain, Injuries, Environmental illness, Disaster epidemiology, Emergency preparedness

## Abstract

**Background:**

Ice, or freezing rain storms have the potential to affect human health and disrupt normal functioning of a community. The purpose of this study was to assess acute health impacts of an ice storm that occurred in December 2013 in Toronto, Ontario, Canada.

**Methods:**

Data on emergency department visits were obtained from the National Ambulatory Care Reporting System. Rates of visits in Toronto during the storm period (December 21, 2013 – January 1, 2014) were compared to rates occurring on the same dates in the previous five years (historical comparison) and compared to those in a major unaffected city, Ottawa, Ontario (geographic comparison). Overall visits and rates for three categories of interest (cardiac conditions, environmental causes and injuries) were assessed. Rate ratios were calculated using Poisson regression with population counts as an offset. Absolute counts of carbon monoxide poisoning were compared descriptively in a sub-analysis.

**Results:**

During the 2013 storm period, there were 34 549 visits to EDs in Toronto (12.46 per 1000 population) compared with 10 794 visits in Ottawa (11.55 per 1000 population). When considering year and geography separately, rates of several types of ED visits were higher in the storm year than in previous years in both Toronto and Ottawa. Considering year and geography together, rates in the storm year were higher for overall ED visits (RR: 1.10, 95 % CI: 1.09-1.11) and for visits due to environmental causes (RR: 2.52, 95 % CI: 2.21-2.87) compared to previous years regardless of city. For injuries, visit rates were higher in the storm year in both Toronto and Ottawa, but the increase in Toronto was significantly greater than the increase in Ottawa, indicating a significant interaction between geography and year (RR: 1.23, 95 % CI: 1.16-1.30).

**Conclusions:**

This suggests that the main health impact of the 2013 Ice Storm was an increase in ED visits for injuries, while other increases could have been due to severe weather across Ontario at that time. This study is one of the first to use a population-level database and regression modeling of emergency visit codes to identify acute impacts resulting from ice storms.

## Background

The U.S. National Weather Service defines an ice storm as an occasion when freezing rain results in damaging accumulation of at least 0.25 in. (6.4 mm) of ice on exposed surfaces [1]. Changnon et al. [2] described 87 ice storms that have occurred in the United States from 1947 to 2000. In Southern Ontario, Klaassen et al. [3] identified 25 ice storms that occurred between 1844 and 2002, the most severe being the 1998 ice storm which led to 28 deaths in eastern Canada and 19 in the northeastern United States.

These storms have the potential to affect human health directly and indirectly, prompting efforts to examine their economic, social and health-related impacts. For example, the 1998 ice storm resulted in temporary consequential impacts on critical infrastructure (i.e. shutting down of transportation, restriction of emergency services and damage to trees and personal property) [4]. Damage from an ice storm is related to ice accumulation adding weight to such structures as tree branches and hydro poles which, when they bend or collapse, result in system disruptions and power outages. From historical evidence in southern Ontario, the potential for an extensive power outage is likely when freezing rain exceeds 40 mm, although even 30 mm of freezing rain may result in severe impact [3].

Some studies on ice storms have investigated acute traumatic injuries such as falls, motor vehicle collisions, injuries from clean-up of debris, and overall rates of fractures and other musculoskeletal injuries [5–9]. Studies from U. S. Centers for Disease Control and Prevention (CDC) and from the Laboratory Centre for Disease Control in Canada (LCDC) (now part of the Public Health Agency of Canada) have examined outcomes such as gastrointestinal illness, cold-related injuries, acute cardiovascular events, respiratory illness and acute psychiatric illnesses [10, 11]. Cardiac events in particular were increased in both of these studies, with a proportional increase in events of 4.2–4.6 % in the CDC study, and a 3.4 fold increase in cardiac events in the LCDC study, comparing ice storm periods with prior year counts.

Carbon monoxide (CO) poisoning is a commonly reported health outcome in ice storm studies. Incidents of CO poisoning are often linked to the use of CO-emitting equipment and appliances in enclosed or semi-enclosed airspaces for the purposes of generating heat or cooking during power outages. Case reports and studies using emergency department (ED), acute care [5, 12–17] and poison control centre data [18] consistently report higher than expected rates of CO poisoning during ice storms, with some studies noting that the onset of cases takes place after a variable lag period following major power outages [12–14, 18].

From December 21 to 23, 2013, the City of Toronto, Ontario, Canada experienced a severe winter storm (2013 Ice Storm) consisting of freezing rain, ice pellets and snow, resulting in ice accumulation and widespread power outages. The increased demand for first responders included 316 calls to Toronto Fire Services for CO exposures and 1 100 calls (63 % higher than expected, based on previous years) to emergency medical services (EMS) for general medical issues, slips and falls and CO exposures [19]. About 300 000 hydro customers in Toronto (approximately 1 000 000 people) lost power for three days, and tens of thousands lost power for more than one week [20]. Various potential hazards were initially identified as a consequence of the ice storm, including absence of heating in homes, lack of refrigeration, slippery sidewalks, falling ice and tree debris, downed live power lines, and use of inappropriate heating sources and generators in enclosed spaces [21].

The purpose of this study was to examine a large population-level database of ED visits in Ontario to identify trends in injury and illness during and immediately following the 2013 Ice Storm, and to determine whether differences particular to the 2013 Ice Storm period exist. Our study evaluated the health impacts of the 2013 Ice Storm on Toronto residents by investigating ED visits during the storm period using two comparisons: a) rates of ED visits in Ottawa during the same time period and b) 5 year historical data for Toronto for the same dates.

## Methods

### Setting

This study compared all ED visit data from two major Canadian municipalities. Toronto, Ontario, the largest metropolitan area in Canada, has a population of about 2.8 million. Ottawa, Ontario, located approximately 350 km from Toronto, is Canada’s capital with a population of 930 000.

### Data sources

Data on ED visits were obtained from the National Ambulatory Care Reporting System (NACRS). All Ontario hospitals report ED data directly to the Canadian Institute for Health Information (CIHI) where the information is compiled and maintained in NACRS. NACRS does not contain detailed demographic information. Population denominator data were obtained from the Ontario Ministry of Health and Long-Term Care (MOHLTC).

### Study design

Rates of ED visits in Toronto during the storm period were compared to a) rates at the same time in Ottawa (geographical comparison) and, b) rates that occurred on the same dates in the previous five years (historical comparison).

The storm period was defined as December 21, 2013, the date the storm began, to January 1, 2014, the date by which all Toronto residents had their power restored. January 1 was also chosen to capture lag effects (e.g. food spoilage causing gastroenteritis) in the greater study population. The geographic boundaries of Toronto and Ottawa were defined using both municipalities’ departments of public health service boundaries in order to ensure consistent inclusion of relevant postal code data. Ottawa was chosen as a geographical comparison because, while less populous than Toronto, it is a large urban centre that experienced snowfall rather than an ice storm. On December 20, 21 and 22, Toronto experienced 12.4 mm, 35.6 mm and 3.6 mm of rain, respectively, with no snow and mean temperatures ranging from 0 to −1.5° Celsius. By comparison, Ottawa experienced 22.8, 4.2 and 14.4 cm of snow with a total of 4.6 mm of rain over the same three days, and mean temperatures from −4.1 to −6.9° Celsius. This infers that Ottawa experienced snow while in Toronto the precipitation was freezing rain. We found no severe storms that occurred during the corresponding dates for the previous five years in either city.

Rates were calculated and compared for overall ED visits and for three disease categories of interest, as selected by consensus of the authors and based on a literature review: cardiac conditions, environmental causes and injuries. Cardiac conditions included cardiac arrest and ischemic heart diseases. Environmental causes included cold exposure, electrical exposure, poisoning from CO and other substances, and heat exposures. Injuries included all primary diagnoses of anatomical injury as well as burns/corrosions, falls, injuries due to tools, and injuries due to vehicles. The categories of interest were selected because of their causal association with this extreme weather event. They were defined according to the International Classification of Diseases, tenth revision (ICD-10). Codes were determined to reflect cardiac conditions [cardiac arrest (I46), ischaemic heart diseases (I20-25)], environmental conditions [cold exposures (T68, T69, T33-35, X31, X37.2), electrical exposures (T75, W85-87), CO/gas poisoning (T57, T58, X47, X49), heat exposure (W92, X00-09)] and injuries [injuries (S00-99, T00-14), burns (T20-32), falls (W00-19), struck by/against (W20-23, W27, W29), vehicles (V01-89)].

Ethics approval for this study was obtained from the Public Health Ontario Ethics Review Board (ID: 2014–026.01).

### Data analysis

Crude rates of overall ED visits per 1 000 population and crude rates of ED visits by disease category per 100 000 population were calculated for the storm period (December 21, 2013 to January 1, 2014) and for the same dates (December 21 to January 1) in the previous five years (2008–09 to 2012–13) in Toronto and Ottawa.

Poisson regression was used to compare ED visits by year and geography using the GENMOD procedure in SAS® version 9.3. Counts of ED visits were used as the dependent variable, year and geography were used as independent variables, and population counts were included as an offset. Four models were fitted for overall ED visits and for each disease category:

- *Model 1*: To answer the question: “were rates of ED visits higher during the Ice Storm in Toronto than they were in Ottawa at the same time?”, rates of ED visits that occurred during the storm period in Toronto (December 21, 2013 to January 1, 2014) were compared to rates that occurred during the same period in Ottawa.
- *Model 2*: To answer the question: “Were rates of ED visits higher during the Ice Storm in Toronto than they were on the same dates in previous years in Toronto?”, rates of ED visits that occurred during the storm period in Toronto (December 21, 2013 to January 1, 2014) were compared to rates that occurred in the same period in Toronto in the previous five years (2008–09 to 2012–13).
- *Model 3*: To answer the question: “Were rates of ED visits higher in Ottawa during the dates the Ice Storm occurred than they were on the same dates in previous years in Ottawa, even though Ottawa did not experience the Ice Storm?”, rates of ED visits that occurred during the storm period in Ottawa (December 21, 2013 to January 1, 2014) compared to rates that occurred in the same period in Ottawa in the previous five years (2008–09 to 2012–13).
- *Model 4*: To assess the joint impact of geography and time, rates of ED visits that occurred during the storm period (December 21, 2013 to January 1, 2014) were compared to rates that occurred during the previous five years (2008–09 to 2012–13) for both Toronto and Ottawa. The presence of an interaction between year and geography was also assessed. Age- and sex-adjusted rates were calculated but were not significantly different from unadjusted rates (not presented).

### Sub-analysis of CO data

CO poisoning was accounted for under the broader category of environmental illnesses. Since the reviewed literature specifically identified CO poisoning as an important and common health impact [5, 12–18], an a priori decision was made to separately compare CO poisoning rates between municipalities and between time periods. After examination of raw data, we determined that counts of CO poisoning were too few outside of the 2013 Ice Storm period to accurately model as a separate variable using Poisson regression analysis. We therefore decided that in addition to the aforementioned models, counts and rates of CO poisoning would be extracted and included as descriptive data without inferential statistical analysis.

## Results

In 2013, although the population of Toronto was about three times that of Ottawa, the two cities had similar age and sex distributions (Table 1).

**Table 1:** Basic demographic characteristics of Toronto and Ottawa in 2013

	Toronto		Ottawa	
	Number	Per cent	Number	Per cent
Population size	2 771 770	N/A	934 300	N/A
Sex				
Female	1 429 844	51.59	477 802	51.14
Male	1 341 926	48.41	456 498	48.86
Age group				
0–19	560 524	20.22	208 985	22.37
20–34	670 246	24.18	209 576	22.43
35–64	1 139 140	41.10	386 626	41.38
65+	401 860	14.50	129 113	13.82

During the 2013 Ice Storm, rates of overall ED visits were 12.46 per 1 000 in Toronto and 11.55 per 1 000 in Ottawa (Table 2). This was higher than any rate observed on the same dates in the previous five years in Toronto, and the second highest rate observed in Ottawa. Rates of ED visits for cardiac conditions were lower during the ice storm than on the same dates in the previous five years for both Toronto (10.64 per 100 000) and Ottawa (11.24 per 100 000).

**Table 2:** Numbers and rates of ED visits between December 21 and January 1, by year

	Toronto		Ottawa	
Overall ED visits	Number	Rate per 100 000	Number	Rate per 100 000
2013–14*	34 549	1 246.46	10 794	1 155.30
2012–13	33 935	1 237.70	10 925	1 181.84
2011–12	31 022	1 147.00	9 716	1 065.06
2010–11	32 135	1 200.79	9 344	1 039.36
2009–10	27 654	1 041.83	8 523	964.42
2008–09	27 279	1 036.30	8 087	930.59
Cardiac conditions				
2013–14*	295	10.64	105	11.24
2012–13	360	13.13	113	12.22
2011–12	329	12.16	110	12.06
2010–11	399	14.91	112	12.46
2009–10	383	14.43	100	11.32
2008–09	420	15.96	150	17.26
Environmental causes				
2013–14*	263	9.49	75	8.03
2012–13	87	3.17	27	2.92
2011–12	89	3.29	27	2.96
2010–11	97	3.62	34	3.78
2009–10	86	3.24	32	3.62
2008–09	132	5.01	38	4.37
Injuries				
2013–14*	6 762	243.96	1 855	198.54
2012–13	5 108	186.30	1 799	194.61
2011–12	5 325	196.89	1 888	206.96
2010–11	4 812	179.81	1 654	183.98
2009–10	5 067	190.89	1 641	185.69
2008–09	5 603	212.85	1 712	197.01

*storm year

In Toronto, rates were higher for ED visits for environmental causes (9.49 per 100 000) and injuries (243.96 per 100 000) during the ice storm than on the same dates in the previous five years. In Ottawa, rates of environmental causes were higher during the storm period (8.03 per 100 000) but the rates of injuries were surpassed in 2011–12.

Table 3 contains rate ratios generated from the four Poisson regression models.

**Table 3:** Rate ratios of ED visits occurring between December 21 and January 1

	Rate ratio	95 % CI
Model 1: Toronto in the storm year compared to Ottawa in the storm year		
Overall ED visits	1.08*	1.06-1.10
Cardiac conditions	0.95	0.76-1.18
Environmental causes	1.18	0.91-1.53
Injuries	1.23*	1.17-1.29
Model 2: Toronto in the storm year compared to Toronto in the previous 5 years		
Overall ED visits	1.10*	1.09-1.11
Cardiac conditions	0.75*	0.67-0.85
Environmental causes	2.59*	2.23-3.01
Injuries	1.26*	1.23-1.30
Model 3: Ottawa in the storm year compared to Ottawa in the previous 5 years		
Overall ED visits	1.11*	1.09-1.14
Cardiac conditions	0.86	0.70-1.06
Environmental causes	2.28*	1.73-3.00
Injuries	1.03	0.97-1.08
Model 4: Storm year compare to the previous 5 years for both Toronto and Ottawa		
Overall ED visits	1.10*	1.09-1.11
Cardiac conditions	0.78*	0.70-0.87
Environmental causes	2.52*	2.21-2.87
Injuries	1.23**	1.16-1.30

*statistically significant difference

**statistically significant interaction

### Model 1: Toronto in the storm year compared to Ottawa in the storm year

During the ice storm, rates of overall ED visits were 8 % higher in Toronto than Ottawa (RR: 1.08, 95 % CI: 1.06-1.10) and rates for injuries were 23 % higher in Toronto than Ottawa (RR: 1.23, 95 % CI: 1.17-1.29). For cardiac conditions and environmental causes, rates were not significantly different between Toronto and Ottawa.

### Model 2: Toronto in the storm year compared to Toronto in the previous five years

Rates of overall ED visits were 10 % higher in Toronto during the ice storm (RR: 1.10, 95 % CI: 1.09-1.11) compared to the same dates from the previous five years in Toronto. For environmental conditions, rates of ED visits were 2.6 times higher (RR: 2.59, 95 % CI: 2.23-3.01) and rates of ED visits for injuries were 26 % higher (RR: 1.26, 95 % CI: 1.23-1.30). Conversely, rates for cardiac conditions were 25 % lower in Toronto during the ice storm (RR: 0.75, 95 % CI: 0.67-0.85) compared to the same dates in Toronto in the previous five years.

### Model 3: Ottawa in the storm year compared to Ottawa in the previous five years

Although Ottawa did not experience the ice storm, during the storm period, rates of ED visits were 11 % higher in Ottawa (RR: 1.11, 95 % CI: 1.09-1.14) compared to the same dates in the previous five years. For environmental conditions, rates of ED visits were 2.3 times higher (RR: 2.28, 95 % CI: 1.73-3.00). There was no significant difference in rates of ED visits for cardiac conditions or injuries.

### Model 4: Toronto in the storm year compared to Toronto in the previous 5 years and Ottawa in the storm year and previous five years

There was no significant interaction between geography and year for overall ED visits, environmental causes or cardiac conditions. Considering geography and year together, rates were higher for overall ED visits (RR: 1.10, 95 % CI: 1.09-1.11) and environmental causes (RR: 2.52, 95 % CI: 2.21-2.87) during the ice storm, regardless of geography. For cardiac conditions, rates of ED visits were 22 % lower during the ice storm (RR: 0.78, 95 % CI: 0.70-0.87), regardless of geography. For injuries, there was a significant interaction between geography and year. Over the time period of the study the risk of injuries increased in both Toronto and Ottawa; however, the increase in Toronto was 23 % higher (RR: 1.23, 95 % CI: 1.16-1.30) than in Ottawa.

### Sub-analysis of CO poisoning

Ninety-eight cases of CO poisoning (3.54 cases per 100 000 population) were diagnosed in Toronto EDs during the 2013 Ice Storm compared with only 1 case (0.11 cases per 100 000 population) of CO poisoning in Ottawa. The numbers of cases over the preceding five-year period ranged from 2–7 per year (0.07 to 0.27 cases per 100 000 population), and 0–2 per year (0 to 0.22 cases per 100 000 population) for Toronto and Ottawa respectively.

## Discussion

The findings from this study suggest that there was an increase in rates of several categories of ED visits between December 21, 2013 and January 1, 2014. Toronto and Ottawa experienced a similar increase in rates of overall ED visits and those due to environmental causes compared to previous years. For injuries, both Toronto and Ottawa experienced an increase in ED visits during this time, but the increase was significantly greater in Toronto than Ottawa. Ottawa was chosen as the comparison city as Environment Canada weather data and media reports indicated that Ottawa was not affected by the same weather pattern as Toronto. As such, it was understood that Ottawa did not experience an ice storm or the accompanying significant power outages [22, 23]. This suggests that the main health impact seen in EDs associated with the 2013 Ice Storm was an increase in visits for injuries, whereas the other increases were more likely due to general severe winter weather experienced in Ontario at that time.

The decision to focus on cardiac events, injuries and environmental causes of illness was based on our literature review on the potential excesses of acute health impacts of ice storms [9–11]. We did not examine injury data by injury site because of the low number of events in each category. Other studies that have examined acute injury data identified a variety of injury types such as falls [5, 6], soft tissue injuries and fractures [7, 8, 11], motor vehicle injuries [13] and injuries related to the recovery process and clean-up [5, 6, 13].

For both Toronto and Ottawa, ED visits due to cardiac causes occurred less frequently than comparison periods. The reason for this is unclear. Both the CDC study [10] and the LCDC study on the 1998 ice storm [11] demonstrated increases in the proportion of visits due to cardiac disease (myocardial infarctions, cardiac arrests, angina). While we expected rates of cardiac events to rise related to strenuous physical activity and emotional stress, it is possible that at-risk individuals avoided precipitating activities because of the severity of the weather system and its aftermath or for other reasons.

With regard to environmental causes of illness, cases of CO poisoning are often cited as one of the most notable findings associated with ice storms as a result of power failures that occur which may prompt the use of other temporary heat sources. In fact, some ice storm studies have focused exclusively on this outcome [15–18, 24]. For this study, the rarity of cases occurring in periods other than the 2013 Ice Storm (i.e., zero cases in most years) prevented the appropriate use of Poisson models to generate comparative statistics. Despite this, the increase in absolute counts for the category of CO poisoning appears convincing. This finding is in keeping with previous studies and serves as a useful reminder of the need to increase surveillance for such events through multiple sources of information (e.g. poison centres, fire service calls, ambulance call reports). Indeed, CO poisoning is likely underreported in other reviews of emergency department data [25], and the City of Toronto noted a five-fold increase in the number of CO-related calls to Fire Services during the 2013 Ice Storm [19].

Ice storms are not necessarily unique in this regard as increases in rates of CO poisoning have been reported from other extreme weather events creating power outages, such as snowstorms [26, 27], hurricanes [25, 28] and floods [25]. This finding also suggests that further emphasis should be placed on prevention and mitigation efforts, such as engineering modifications (e.g. adding auto shut-offs to generators and other CO-producing devices), better policies requiring CO alarm installations, and enhanced pre-emergency/disaster risk communication that is effective for a variety of audiences [25, 29]. These could also include public announcements and warning labels to increase awareness of the risks of using poorly maintained or located generators, starting keyless cars in garages and bringing charcoal grills indoors [25].

The design used in this study differs substantially from the majority of literature on health impacts of ice storms. First, it employs the use of population-level data, which appears to have only been employed in one other study describing an ice storm in Oklahoma in 2007 [13]. However, the Oklahoma study was limited to assessment of mandatory data reports that were reported solely for the purposes of tracking information during the storm itself, and thus no control was captured for comparison. The LCDC Study [11] compared findings from the 1998 ice storm in Eastern Ontario with comparable periods in the preceding year, but this data was gathered through chart review data from five eastern Ontario hospitals over a four-week period comparing the 1998 storm year and the previous year. The use of data gleaned from chart reviews is a reasonable approach but is limited by chart selection as well as questions about the rigour and consistency of data extraction methods. By comparison, our study made use of NACRS, an existing robust administrative population-based database; as such, the data were not opportunistically gathered for the purposes of this study, but rather were collected contemporaneously, cleaned and maintained. We are thus confident in the data’s scope, quality and objectivity.

Second, as the data represented counts occurring during specific periods of time, the use of Poisson regression was an improvement upon previous analyses of ice storm impacts, as other studies often provided only descriptive statistics or very basic comparisons with control periods. However, as discussed previously, quantitative analyses may not reveal rare but consequential events such as CO poisoning.

### Limitations

This study made use of both diagnostic codes and codes that specify external causes using ICD nomenclature and coding. The accuracy of the data captured by NACRS is reflective of how care providers and hospital coders identified both accurate diagnoses and external causes of relevance (e.g. cold exposures, CO exposures). Since the dataset used in this study was of sufficient size and represented the entire City of Toronto and City of Ottawa, and since the NACRS database is considered to be high quality [30], it is unlikely that inaccurate coding would have had a significant effect on the results produced. Moreover, our selection of ICD codes, although informed by existing literature, may have missed some health impacts attributable to this event.

A major limitation of this study is that although the data used represents individual-level outcomes, the exposure in question is defined by geography and time at the population-level. This limits the degree to which one can establish a causal relationship between the ice storm and the health impacts of interest. Considering the nature of ice storms, it is unlikely that an individual criterion could serve as a marker of exposure to the effects of an ice storm. Thus, population-level attribution may be a useful and feasible approach for studying such events in the post-event phase. Disaster epidemiology designs, such as rapid needs assessments, will continue to provide important information in public health response planning [31]. Given this limitation, we attempted to improve the robustness of our analysis by using both geographic and historic comparisons subject to similar climate conditions, the same dates of the year and similar population milieu to demonstrate the differences seen. The fact that rates of ED visits were found to be significantly elevated during the 2013 Ice Storm period despite analyzing a large volume of population-level data over multiple years supports the possibility that these higher visit counts and rates may have been due to the short-term impact of the weather system in Ontario, although the degree to which the ice storm created an additional overall burden was not clear. It is of interest to note that during the study periods heavy precipitation occurred in Toronto and Ottawa in 2008–9 (mixed), 2009–10 (rain) and 2012–13 (snow). An increase in ED visits in the three categories occurred only in 2008–9. The reason for this is unclear, but it is possible that mixed precipitation with temperatures straddling the freezing point may have led to more physical and environmental injuries compared to rain or snow. Unfortunately we were unable to access detailed information about power outages during these periods. The finding of a significant increase in environmental illness ED visits compared to previous years in both Toronto and Ottawa is curious. The weather system that produced the 2013 Ice Storm in Toronto also affected Ottawa to some degree; hence it is possible that Ottawa experienced some of the effects of severe weather without the larger effect on injuries that we have attributed to the specific effects of ice. Accordingly, municipal cold weather plans should address both direct and indirect impacts of cold weather events which include the impacts of snow, ice and power outages.

Another major limitation of this study is that the data do not reflect health impacts in a broad sense but rather one component of health system utilization. In the absence of comprehensive data on illnesses and injuries, rates of ED visits were used as a proxy. We note that by focusing on health services through ED visits, other acute health impacts might have been missed, such as those identified through home care, non-ED primary care and social services. As well, the subacute and chronic health impacts from ice storms are not explored in this study, nor are broader social determinants of health. This is due the fact that our source of data only reflects episodic involvement of health care offered through ED visits. Other authors have examined longer-term outcomes from ice storms such as effects on pre-term birth [32] and latent outcomes influenced by prenatal maternal stress [33]. In addition to the limitations of ED visit data, the demographic information available for these visits is limited to sex and age, which prevented further examination of particular social and economic vulnerabilities that might differentially impact health in this context.

## Conclusions

Our study has demonstrated an increase in ED visits for injuries in Toronto during the 2013 Ice Storm, which suggests that the greatest health impact from the storm, as seen in EDs, was injuries. Rates of overall ED visits, and ED visits related to environmental conditions increased during the storm period, but this increase was found in both Toronto and Ottawa. On the basis of our findings municipal cold weather plans should take into account the health impacts of ice storms, including physical and environmental injuries.

This study is one of the first to use systematic population-level data and regression modelling of emergency care visit codes to identify acute impacts resulting from ice storms. It provides validation and support of anecdotal and descriptive reports in the published literature on the effects of ice storms, and can be used by health emergency personnel to plan for ED and in-hospital surge capacity for these events. It can also inform public health messaging on the threats and mitigation of physical and environmental injuries. Our findings do not fully describe and possibly underestimate the health impacts that the 2013 Ice Storm had on the City of Toronto. As extreme weather events are likely to increase in frequency and severity due to climate change [34], future studies in disaster epidemiology may benefit from taking a similar approach using population-level data to assess health impacts from these large-scale events.

## Abbreviations

CDC, Centers for disease control and prevention; CI, Confidence interval; CIHI, Canadian institute for health information; CO, Carbon monoxide; ED, Emergency department; ICD, International classification of diseases; LCDC, Laboratory centre for disease Control; MOHLTC, Ontario ministry of health and long-term care; NACRS, National ambulatory care reporting system; RR, Risk ratio
